# Effectiveness of interventions to support the transition home after acute stroke: a protocol for a systematic review

**DOI:** 10.12688/hrbopenres.13364.1

**Published:** 2021-09-23

**Authors:** Geraldine O'Callaghan, Martin Fahy, Paul Murphy, Peter Langhorne, Rose Galvin, Frances Horgan

**Affiliations:** 1 RCSI School of Physiotherapy, Royal College of Surgeons in Ireland, Dublin 2, DO2 YN77, Ireland; 2 Division of Population Health Sciences, Royal College of Surgeons in Ireland, Dublin, Ireland; 3 RCSI Library, Royal College of Surgeons in Ireland, Dublin, Ireland; 4 Institute of Cardiovascular and Medical Sciences, University of Glasgow, Glasgow, G31 2ER, UK; 5 School of Allied Health, Faculty of Education and Health Sciences, Ageing Research Centre, Health Research Institute, University of Limerick, Limerick, V94 T9PX, Ireland

**Keywords:** Stroke, Transition, Systematic review, Intervention

## Abstract

Background

Despite advances in the quality of acute stroke management, there are gaps in knowledge about effective support interventions to better manage the transition of care to home for patients with this complex condition.  The goal of this systematic review is to explore the literature around support interventions available for patients as they navigate from acute hospital, rehabilitation or early supported discharge (ESD) services to independent living at home; and to establish if, in comparison with usual care or other comparative active interventions, support services offered to patients as they transition from acute hospital, inpatient rehabilitation/ESD to home, can achieve better patient and / or process outcomes.

Protocol

In September 2021, we will carry out, on electronic peer-reviewed databases, a comprehensive literature search based on a pre-defined search strategy, developed and conducted in collaboration with an Information Specialist.   In an effort to identify all published trials we will perform citation tracking of included studies, check reference lists of relevant articles, review grey literature, and extend our search to google scholar.

We will include randomised controlled trials (including cluster and quasi-randomisation) recruiting stroke patients transitioning to home, to receive either usual care or any support intervention designed to improve outcomes after stroke.

The primary clinical outcome will be functional status.  Two review authors will scrutinise trials, categorise them on their eligibility, and extract data. We will analyse the results for all trials and perform meta-analyses where possible.  We will assess risk of bias for the included trials and use GRADE to assess the quality of the body of evidence.

Patient and public involvement (PPI) engaged in the development of the research questions, and will participate in co-design of a strategy for dissemination of findings.

**Conclusions: **The findings from this review will be used to identify knowledge gaps to direct future research.

## Introduction

Stroke is one of the leading causes of death and disability worldwide ^1^. While incidence, prevalence, mortality, and disability-adjusted life-years rates are in decline, “the overall stroke burden in terms of absolute number of people affected by, or who remained disabled from, stroke has increased across the globe in both men and women of all ages” ^2^. Costs of stroke are rising, partly due to an ageing population, and the economic burden of stroke across Europe is currently estimated at 60 billion ^3^. 

Rehabilitation interventions such as early supported discharge (ESD) reduce hospital lengths of stay and costs in stroke care, while improving outcomes such as physical, cognitive and psychosocial function ^4^. However, patients recovering from acute stroke have complex needs and face significant challenges in self-management of hospital to home transitions ^5^. The American Geriatrics Society defines transitions of care as: “a set of actions designed to ensure the coordination and continuity of health care as patients transfer between different locations or different levels of care” ^6^. Interventions at transitions of care are recognised as key to care coordination, impacting on quality of care and adverse events ^7^. 

Stroke survivors, caregivers and healthcare professionals identify a focus on minimising stroke-related impairments, adjusting to life after stroke, and receiving information and guidance in relation to the long-term consequences of stroke and secondary stroke prevention, as research priorities ^8^. Stroke survivors require signposting in order to navigate the healthcare system, and information and support in relation to healthy living, fatigue management, maintaining physical activity, managing stress and psychological issues, return to work and driving ^9– 11^. 

There is an opportunity for “support” interventions, provided when individuals are discharged from hospital or inpatient rehabilitation to home, to improve continuity and quality of care, improve functional outcomes, reduce healthcare utilisation, and improve patient and carer experience ^12^, but there are gaps in knowledge about effective “support” interventions to better manage transitions for this complex health condition. A systematic review by Prvu Bettger *et al.*, 2012, of the benefits or harms of interventions at transitions of care after hospitalisation for stroke or myocardial infarction, showed low-to-moderate strength evidence of effectiveness of hospital-initiated transitional care, but insufficient evidence for education, community-based models of support, and chronic disease management models of care for patients with stroke or myocardial infarction ^13^. Wang *et al.*, 2017, sought to identify the type of interventions at transitions of care to effectively reduce mortality and improve activities of daily living (ADLs) in stroke patients, categorising supports at transitions of care into hospital-initiated support, home-visiting programmes, telephone support, primary education, and out-patient setting support. Their meta-analysis showed that only home-visiting programmes reduced mortality rates (Risk Ratio (RR)=0.46; 95% Confidence Interval (CI): 0.28-0.74), and impacted positively on patient ADLs (RR=0.56; 95% CI: 0.31-0.81) after stroke, compared with usual care ^14^. 

Given the time since the most recent review ^14^, the subsequent publication of a number of transitions of care intervention studies ^15, 16^, and policy plans to shift the way in which health and social care services are delivered ^17^, we determined a systematic review of effectiveness of “support” interventions provided at transitions of care after stroke to be feasible.

An evidence synthesis will aid in identifying the key “support” elements that promote successful hospital-home transitions for stroke survivors and their families/caregivers. The aim of this protocol is to describe the methods we will employ to synthesise the best available evidence in relation to the effectiveness of support interventions provided at transitions of care after stroke on clinical and process outcomes.

## Protocol

Design: We will conduct a systematic review of randomised controlled trials (including cluster and quasi-randomisation) exploring the effectiveness of supports provided at transitions of care (moving between acute hospital/inpatient rehabilitation and home) after stroke. This protocol has been prepared in accordance with the Preferred Reporting Items for Systematic Review and Meta-Analysis Protocol (PRISMA-P) statement and checklist ^18, 19^, and it is registered in PROSPERO.

### Stage 1: Identifying the research question

The PICOTS (population, intervention, comparison, outcomes, types of study design, setting) acronym has been applied to clarify the research question.

**•  Population**

Adult stroke survivors (18 years of age or older) discharged from acute hospitalisation, inpatient rehabilitation to home. Studies that include TIA or subarachnoid haemorrhage will be accepted if > 80% of the participants have had an ischaemic stroke.

**•  Intervention(s)**

“Support” intervention will be defined as an intervention specifically designed to help facilitate stroke survivors to self-manage their care after discharge from acute hospitalisation or inpatient rehabilitation to home e.g. individualised discharge plan, patient booklet, stroke passport etc. Interventions can take place before or after discharge, or include bridging interventions i.e. components that span settings (e.g. home visit from the acute setting). We will exclude an evidence synthesis of ESD interventions from the acute setting as this has been completed ^4, 20^. However, studies that explore interventions to bridge the transition to the community following ESD will be included.

**•  Comparator(s), Control**

Usual care or other active interventions e.g. individualised discharge plan and patient booklet versus patient booklet.

**•  Outcomes**

The primary outcome of interest is functional status. All validated outcomes of functional status will be considered e.g. Functional Independence Measure (FIM); Barthel Index (BI). 

Secondary outcomes will include clinical (physical and psychological) and process (emergency department visits, rehospitalisation, mortality) outcomes assessed within one year of hospital discharge. 

Adverse events, expected (e.g. scheduled medical or surgical procedures) and unexpected (e.g. recurrent stroke, seizure, falls), will also be examined.

### Stage 2: Identifying relevant studies

Randomised controlled trials (including cluster and quasi-randomisation) will be included. Studies included in quasi-randomisation will be limited to those that have a “strong” design, as determined by a checklist of design features ^21^, which can estimate cause and effect with minimal risk of bias. 

A systematic review of the literature will be conducted, with three groups of keywords related to “stroke”, “transition”, and “care setting”. One reviewer (GOC) will complete a comprehensive search of electronic databases ( Cochrane Central Register of Controlled Trials, MEDLINE, EMBASE, CINAHL, Cochrane Library, APA PsychInfo and SCOPUS), based on a pre-defined search strategy, developed and conducted in collaboration with an Information Specialist (PM). The search will initially be conducted in MEDLINE via OVID, and we will then adapt this strategy to other databases. Databases will be searched from inception, with no language limitation. The reference list of articles that meet the inclusion criteria after full-text review will be hand-searched to identify additional articles. We will include a search of grey literature, and extend the search to Google Scholar. We will attempt to contact authors of published abstracts to request full-text versions of studies and/or study data. The full search strategy, with MESH and Keywords can be found in Table 1. 

**Table 1.** Search Strategy for Ovid Medline, COCHRANE LIBRARY and CENTRAL REGISTRY OF CLINICAL TRIALS on Wiley, EMBASE on Elsevier.com, CINAHL on Ebscohost, APA PsychINFO on Ebscohost and SCOPUS on Elsevier.com. What is the effectiveness of support interventions provided at transitions of care after stroke on clinical and process outcomes?

	Ovid MEDLINE(R)
1	Stroke.mp. OR exp Stroke/ OR (Post stroke).mp. OR poststroke.mp. OR Stroke rehabilitation.mp. OR exp Stroke Rehabilitation/ OR Cerebro-vascular accident.mp. OR (Cerebro vascular).mp. OR CVA.mp. OR exp cerebrovascular disease/ OR (cerebrovascular adj1 disease).mp.
2	subarachnoid hemorrhage.mp. or Subarachnoid Hemorrhage/
3	1 NOT 2
4	(Continuity adj3 patient adj3 care).mp. OR exp Continuity of Patient Care/ OR (patient adj2 discharge).mp. or exp Patient Discharge/ OR (discharge adj1 plan*).mp.
5	(Transition* adj2 care).mp. OR exp Transitional Care/ OR (Patient adj2 transition*).mp. OR (Patient adj2 handoff).mp. OR (Patient adj2 transfer).mp. OR exp Patient Transfer/
6	Exp Patient Navigation/ OR (Patient adj2 Navigation).mp. OR *Case Management/ OR (Posthospital OR Post hospital OR Post discharge).mp.
7	4 OR 5 OR 6
9	Exp Home Care Services/ OR (home adj3 care).tw. OR Exp Home Care Services, Hospital-Based/ OR Exp Home Nursing/ OR Exp Primary Health Care/ OR (primary adj2 care).mp. OR (domiciliary adj 1 care).mp. OR (Community adj2 based adj2 support$) OR Exp Community Health Services/ OR (community adj1 care).mp.
10	3 AND 7 AND 9

**Table:** 

	COCHRANE LIBRARY and CENTRAL REGISTRY OF CLINICAL TRIALS on Wiley
1	Mesh Descriptor: [stroke] explode all trees OR MeSH descriptor: [Cerebrovascular Disorders] explode all trees OR Mesh Descriptor: [Stroke Rehabilitation] explode all trees OR stroke:ti,ab OR poststroke:ti,ab
2	subarachnoid NEAR/1 hemorrhage:ti,ab,kw
3	#1 NOT #2
4	Patient NEAR/1 care:ti,ab,kw OR patient NEAR/2 discharge:ti,ab,kw OR discharge NEAR/1 plan:ti,ab,kw OR Transition NEAR/2 care: ti,ab,kw OR transitional NEAR/1 care OR Patient NEAR/1 transition:ti,ab,kw OR patient NEAR/1 handoff:ti,ab,kw OR Patient NEAR/1 transfer:ti,ab,kw
5	Mesh Descriptor:[Patient Discharge] explode all trees
6	#4 OR #5
7	Mesh Descriptor: [Primary Health Care] explode all trees
8	Home NEAR/1 care:ti,ab,kw OR Domiciliary NEAR/1 care:ti,ab,kw
9	#7 OR #8
	#9 = Cochrane Systematic Reviews
	#9 = Central Registry of Clinical Trials

**Table:** 

	EMBASE on Elsevier.com
1	stroke:ti,ab,kw OR Stroke/exp OR ‘Post stroke’:ti,ab,kw OR poststroke:ti,ab,kw OR ‘Stroke rehabilitation’:ti,ab,kw OR ‘acute ischemic stroke’/exp OR ‘cerebrovascular accident’/exp OR ‘Cerebrovascular accident’:ti,ab,kw OR CVA:ti,ab,kw OR ‘cerebrovascular disease ’:ti,ab,kw
2	‘subarachnoid hemorrhage’:ti,ab OR ‘Subarachnoid Hemorrhage’/exp
3	#1 NOT #2
4	‘Continuity patient care’:ti,ab,kw OR ‘patient discharge’:ti,ab,kw OR ‘hospital discharge’/exp OR discharge:ti,ab,kw OR ‘discharge plan*’:ti,ab,kw
5	‘Transition* care’:ti,ab,kw OR ‘Transitional Care’/exp OR ‘Patient transition*’:ti,ab,kw OR ‘Patient handoff’:ti,ab,kw OR ‘Patient transfer’:ti,ab,kw
6	‘Patient Navigation’:ti,ab,kw OR ‘Case Management’:ti,ab,kw OR (Posthospital OR ‘Post hospital’):ti,ab,kw
7	4 OR 5 OR 6
9	‘Home Care’/exp OR ‘home care’:ti,ab,kw OR ‘Home Nursing’:ti,ab,kw OR ‘Primary Health Care’/exp OR ‘primary care’:ti,ab,kw OR ‘domiciliary care’:ti,ab,kw OR ‘Community based support$’:ti,ab,kw OR ‘community care’:ti,ab,de,kw
10	3 AND 7 AND 9

**Table:** 

	CINAHL on Ebscohost
1	(MH “Stroke+”) OR (MH “Cerebral Hemorrhage+”) OR TI stroke OR AB stroke OR "stroke rehabilitation" OR TX cerebrovascular N1 accident$ OR cerebral N1 hemorrhage
2	subarachnoid N1 hemorrhage
3	S1 NOT S2
4	(MH "Continuity of Patient Care+") OR (MH "Patient Discharge") OR "patient discharge" OR (MH "Transfer, Discharge") OR (MH "Discharge Planning")
5	(MH "Transitional Care") OR "transitional care" OR “Patient transition* OR (Patient handoff” OR “Patient transfer” OR “patient navigation”
7	4 OR 5
8	(MH "Home Health Care") OR "home care" OR “domiciliary care” OR (MM "Primary Health Care") OR (primary N2 care) OR (MH "Community Health Nursing+")
9	3 AND 7 AND 8

**Table:** 

	APA PsychINFO on Ebscohost
1	TX stroke OR TX ‘Cerebrovascular Accidents’
2	TX ‘subarachnoid hemorrhage’
3	S1 NOT S2
4	TX continuity N3 care OR MM "Continuum of Care" OR TX patient N2 discharge OR DE "Hospital Discharge" OR DE "Discharge Planning" OR TX (Transition* N2 care) OR TX Patient* N2 transition OR TX 'Patient handoff' OR TX 'Patient transfer' OR TX Patient N2 navigation OR TX post N1 discharge
5	TX home N1 care OR MM "Home Care" OR TX domiciliarly N1 care OR MM "Primary Health Care" OR TI Primary N3 care OR AB Primary N3 care
6	3 AND 4 AND 5

**Table:** 

	SCOPUS on Elsevier.com
1	TITLE-ABS-KEY ( ( “stroke” ) OR ( “poststroke” ) OR ( "cerebrovascular accident" ) OR ( "Stroke rehabilitation" ) OR ( "Cerebrovascular accident" ) )
2	TITLE-ABS-KEY ( "subarachnoid hemorrhage" )
3	#1 AND NOT #2
4	TITLE-ABS-KEY(“continuity of care” OR “patient discharge” OR “hospital discharge” OR "discharge plan" OR "discharge planing" OR "post discharge” OR “Transitional care” OR “Patient transition” OR “patient handoff” OR “Patient transfer” OR “patient navigation”)
5	TITLE-ABS-KEY(“home care” OR “Home Nursing” OR “Primary Health Care” OR “primary care” OR “domiciliary care”)
6	3 AND 4 AND 5

### Stage 3: Study selection

All articles will be imported into Endnote X9 bibliographic software, and where possible duplicates will be deleted. The primary researcher (GOC) will carry out an initial scan to judge and exclude clearly irrelevant literature. Two reviewers (GOC and RG) will independently review the remaining titles and abstracts from transitional care trials obtained in the literature search and compare them to the inclusion/exclusion criteria. They will meet to reach consensus about full text inclusion. If either one of the reviewers deems that an article could fulfil the inclusion criteria, then the article will be included for full text screening. Two reviewers (GOC and RG) will read each article to confirm eligibility using the same agreement structure as for abstract screening. 

### Stage 4: Charting the data

Characteristics of the intervention such as

1. Study design,
2. Funding source,
3. Setting,
4. Geographic location,
5. Participant characteristics (age, sex, race, ethnicity, severity of stroke, communication status, support system and urban/rural),
6. Intervention characteristics (transitional care component details, theory, comparator, outcome results including adverse events, and time to follow up),
7. Risk of Bias,

will be extracted by one reviewer (GOC) into evidence tables using Rayyan systematic review screening software. The data abstraction will be reviewed by a second reviewer (FH) for accuracy. Disagreements will be resolved by consensus. All studies included will be critically appraised by two independent reviewers (GOC and FH) applying The Cochrane tool for assessing risk of bias version 2 (RoB 2) in randomised trials ^22^. The RoB2 tool covers 5 Domains and risk of bias is categorised as “low risk of bias”, “some concerns” or “high risk of bias”. Disagreements will be resolved through discussions, and if necessary by consulting a third reviewer (RG). Studies will not be excluded based on risk of bias but we will include this assessment in rating the strength of evidence. 

GRADE (Grading of Recommendations, Assessment, Development and Evaluations) provides an explicit framework for rating the quality of evidence in systematic reviews ^23^. The overall certainty in the evidence, based on our confidence that the estimate of effects are correct, will be assessed for each outcome identified across studies using GRADE’s four categories (high, moderate, low, or very low) ^24^.

### Stage 5: Collating, summarising, and reporting the results

Data will be extracted separately for the meta-analysis. For each study, data on the outcomes will be extracted post-intervention and at follow-up time points. For our primary outcome of functional status, data extraction will comprise mean and standard deviation values, across the intervention and control groups as well as the number in each group at baseline. Pooled mean differences (MD) and 95% confidence intervals (CI) will be calculated to determine treatment effect. Pooled risk ratios and 95% CI for dichotomous outcomes will be calculated. If the scale for each continuous outcome varies across the studies, we will calculate a standardised mean difference (SMD) and 95% CI based on end-of-study results. If studies report changed values and the baseline value is not available, we will use this data in meta-analyses but plan sensitivity analyses to investigate the effect of using these data. We will analyse the incidence of adverse events as dichotomous variables.

Meta-analysis will be performed using Review Manager 5 ( RevMan5, Cochrane) ^25^. We will determine heterogeneity using visual inspection of the forest plots and the I ^2^ statistic. In the first instance, we will assume homogeneity across out studies and we will complete our meta-analysis using a fixed effects model and 95% CI. If the I ^2^ reveals > 50% indicating substantial clinical heterogeneity (different interventions) or methodological heterogeneity (variation in risk of bias across studies), then we will compute using a random effect model and 95% confidence intervals. For heterogeneity present after meta-analysis, subgroup analysis will be carried out for potential sources of heterogeneity ^26^.

Where statistical pooling is not possible, the findings will be presented in table and narrative form. 

**•  Missing outcome data**

The extent of missing outcome data will be recorded on our risk of bias table.

Where the reporting of an outcome in a particular study is unclear, incomplete, or missing we will attempt to contact the study’s authors to obtain the data. If we are unsuccessful in obtaining the data, we will not include that study in the meta-analysis of that outcome.

If an included study has missing data (e.g. reports means but not standard deviations for follow-up data) we will attempt to contact the study’s authors to obtain the data. If we are unsuccessful, then we will take logical steps to enter an assumed value, by estimating a standard deviation based on a reported standard error, estimating a follow-up standard deviation based on a baseline value, using the median as a proxy for the mean, and using a multiple of 0.75 times the interquartile range or 0.25 times the range as a proxy for the standard deviation values ^27^. We will undertake a sensitivity analysis to investigate the effect of entering assumed values.

**•  Sensitivity analysis**

*Selection bias*: We will exclude studies with evidence of selection bias (bias in randomisation or allocation concealment processes)

*Missing outcome data*: we will re-analyse the data, excluding trials with inadequate or unclear methods of dealing with missing outcome data.

*Quality/risk of bias*: we will re-analyse the data, excluding trials deemed to be at high risk of bias.

*Quasi-randomised trials*: we will reanalyse to determine how data from studies with quasi-randomisation influence the treatment effect.

**•  Subgroup analyses**

We will attempt to conduct sub-group analyses for intervention types, choice of comparison intervention, and duration of follow up.

### Stage 6: Patient and public involvement (PPI)

The ACTIVE framework ^28^ and GRIPP2-SF ^29^ will help describe and report PPI in the systematic review. Engaging PPI partners will ensure that the systematic review is relevant and meaningful to people affected by stroke, and to people using the review to inform health policy or practice. A “top-and-tail” approach ^29^ will be used to involve the same group of people at the start (stages 1 to 3: framing the question and planning the review) and end (stages 10 to 12: interpretation, publication and dissemination of findings) of the review. A PPI Champion (Stroke survivor (MF)) will contribute as a core member of the review team, while an advisory network comprising stakeholders representing key groups in stroke (patients and their family members, carers, healthcare professionals, advocacy groups) will be formed. Recruitment to the advisory network will take place throughout the review, and different individuals will have varying levels of involvement, and at different stages. 

## Ethical considerations

The systematic review consists of reviewing and collecting data from publicly available materials, and therefore does not require ethics approval. 

The systematic review constitutes the first step in a multi-phased research project aimed at improving and supporting transitions of care after stroke by understanding the long-term needs of stroke survivors; and by identifying the effectiveness of, and preferences, for supports provided at transitions of care.

The results from this systematic review will guide and be combined with data from later phases of the research, including an observational study and qualitative interviews and focus groups with stroke survivors and healthcare professionals, and a co-design process.

Ethics approval will be sought for these later stages of the research. 

### Dissemination

Upon completion, the findings of the systematic review will be published in a peer-reviewed open-source journal; presented at national and international conferences; and shared with researchers, clinicians, stroke survivors and families through organisations for people with stroke. We will provide recommendations for practice and research. PPI will engage in the development of the dissemination strategy, and assist in summarising the research is a clear and accessible format.

### Study Status

The study protocol has been registered on PROSPERO (CRD42021237397).

At the time of publication of this protocol, the database searches will have been completed and screening initiated.

### Tracked and dated amendments

Any amendments to this protocol, including the dates of the amendments and justifications, will be documented and presented in a table in the systematic review publication.

## Conclusions

Given the challenges experienced by stroke survivors during the transition from acute stroke services to home, there is an urgent need to better understand what interventions are effective in supporting this transition. This systematic review will broadly and systematically explore the totality of evidence around support types provided at hospital to home transitions after stroke. Findings will be used to identify knowledge gaps to direct future research and provide a foundation for developing research priorities.

## Data availability

### Underlying data

No data are associated with this article.

### Reporting guidelines

Zenodo PRISMA-P checklist for “Effectiveness of interventions to support the transition home after acute stroke: A protocol for a systematic review”. https://doi.org/10.5281/zenodo.5145362 ^30^

Data are available under the terms of the Creative Commons Attribution 4.0 International license (CC-BY 4.0).

## References

[1] World Health Organization: Global Health Estimates.Geneva;2012. Reference Source

[2] Feigin VL Norrving B Mensah GA : Global burden of stroke. *Circ Res.* 2017;120(3):439–48. 10.1161/CIRCRESAHA.116.308413 28154096

[3] Luengo-Fernandez R Violato M Candio P : Economic burden of stroke across Europe: A population-based cost analysis. *Eur Stroke J.* 2020;5(1):17–25. 10.1177/2396987319883160 32232166 PMC7092742

[4] Langhorne P Baylan S, Early Supported Discharge Trialists : Early supported discharge services for people with acute stroke. *Cochrane Database Syst Rev.* 2017;7(7):CD000443. 28703869 10.1002/14651858.CD000443.pub4 PMC6483472

[5] Chen L Xiao LD Chamberlain D : An integrative review: Challenges and opportunities for stroke survivors and caregivers in hospital to home transition care. *J Adv Nurs.* 2020;76(9):2253–65. 10.1111/jan.14446 32511778

[6] Coleman EA Boult C, American Geriatrics Society Health Care Systems Committee : Improving the Quality of Transitional Care for Persons with Complex Care Needs. *J Am Geriatr Soc.* 2003;51(4):556–7. 10.1046/j.1532-5415.2003.51186.x 12657079

[7] Naylor MD Aiken LH Kurtzman ET : The care span: The importance of transitional care in achieving health reform. *Health Aff (Millwood).* 2011;30(4):746–54. 10.1377/hlthaff.2011.0041 21471497

[8] Pollock A St George B Fenton M : Top 10 research priorities relating to life after stroke--consensus from stroke survivors, caregivers, and health professionals. *Int J Stroke.* 2014;9(3):313–20. 10.1111/j.1747-4949.2012.00942.x 23227818

[9] Sumathipala K Radcliffe E Sadler E : Identifying the long-term needs of stroke survivors using the International Classification of Functioning, Disability and Health.Sumathipala, K., Radcliffe, E., Sadler, E., Wolfe, C. D. A. & McKevitt, C. Mar 2012 In : *Chronic Illness.*8(1): 31-44 14. Research output: Contribution to journal › Article. *Chronic Illn.* 2012;8(1):31–44. 10.1177/1742395311423848 22025770

[10] Walsh ME Galvin R Loughnane C : Factors associated with community reintegration in the first year after stroke: a qualitative meta-synthesis. *Disabil Rehabil.* 2015;37(18):1599–608. 10.3109/09638288.2014.974834 25382215

[11] Miller KK Lin SH Neville M : From Hospital to Home to Participation: A Position Paper on Transition Planning Poststroke. *Arch Phys Med Rehabil.* 2019;100(6):1162–75. 10.1016/j.apmr.2018.10.017 30465739

[12] McElwaine P McCormack J Harbison J : National Stroke Audit 2015.2016. Reference Source

[13] Prvu Bettger J Alexander KP Dolor RJ : Transitional care after hospitalization for acute stroke or myocardial infarction: a systematic review. *Ann Intern Med.* 2012;157(6):407–16. 10.7326/0003-4819-157-6-201209180-00004 22986378

[14] Wang Y Yang F Shi H : What Type of Transitional Care Effectively Reduced Mortality and Improved ADL of Stroke Patients? A Meta-Analysis. *Int J Environ Res Public Health.* 2017;14(5):510. 10.3390/ijerph14050510 28489044 PMC5451961

[15] Reeves MJ Fritz MC Woodward AT : Michigan Stroke Transitions Trial. *Circ Cardiovasc Qual Outcomes.* 2019;12(7):e005493. 10.1161/CIRCOUTCOMES.119.005493 31296043

[16] Duncan PW Bushnell CD Jones SB : Randomized pragmatic trial of stroke transitional care: the Compass Study. *Circ Cardiovasc Qual Outcomes.* 2020;13(6):e006285. 10.1161/CIRCOUTCOMES.119.006285 32475159

[17] Department of Health: Sláintecare Implementation Strategy. 2018. Reference Source

[18] Moher D Shamseer L Clarke M : Preferred reporting items for systematic review and meta-analysis protocols (PRISMA-P) 2015 statement. *Syst Rev.* 2015;4(1):1–9. 10.1186/2046-4053-4-1 25554246 PMC4320440

[19] Shamseer L Moher D Clarke M : Preferred reporting items for systematic review and meta-analysis protocols (PRISMA-P) 2015: elaboration and explanation. *BMJ.* 2015;350:g7647. 10.1136/bmj.g7647 25555855

[20] Langhorne P Taylor G Murray G : Early supported discharge services for stroke patients: a meta-analysis of individual patients' data. *Lancet.* 2005;365(9458):501–6. 10.1016/S0140-6736(05)17868-4 15705460

[21] Reeves BC Wells GA Waddington H : Quasi-experimental study designs series-paper 5: a checklist for classifying studies evaluating the effects on health interventions-a taxonomy without labels. *J Clin Epidemiol.* 2017;89:30–42. 10.1016/j.jclinepi.2017.02.016 28351692 PMC5669452

[22] Sterne JAC Savović J Page MJ : RoB 2: a revised tool for assessing risk of bias in randomised trials. *BMJ.* 2019;366:l4898. 10.1136/bmj.l4898 31462531

[23] Guyatt G Oxman AD Akl EA : GRADE guidelines: 1. Introduction-GRADE evidence profiles and summary of findings tables. *J Clin Epidemiol.* 2011;64(4):383–94. 10.1016/j.jclinepi.2010.04.026 21195583

[24] The GRADE Working Group: GRADE handbook for grading quality of evidence and strength of recommendation. 2013. Reference Source

[25] Cochrane Collaboration: Review Manager 5 (RevMan5). Reference Source

[26] Deeks HJ Altman DG, on behalf of the Cochrane Statistical Methods Group: Analysing data and undertaking meta-analysis.In: Higgins JPT TJ, Chandler J, Cumpston M, Li T, Page MJ, Welch VA (editors),, editor. *Cochrane Handbook for Systematic Reviews of Interventions*. 2nd edition ed. Chichester (UK): John Wiley & Sons; 2019;258–66.

[27] Hozo SP Djulbegovic B Hozo I : Estimating the mean and variance from the median, range, and the size of a sample. *BMC Med Res Methodol.* 2005;5(1):13. 10.1186/1471-2288-5-13 15840177 PMC1097734

[28] Pollock A Campbell P Struthers C : Development of the ACTIVE framework to describe stakeholder involvement in systematic reviews. *J Health Serv Res Policy.* 2019;24(4):245–55. 10.1177/1355819619841647 30997859

[29] Staniszewska S Brett J Simera I : GRIPP2 reporting checklists: tools to improve reporting of patient and public involvement in research. *BMJ.* 2017;358:j3453. 10.1136/bmj.j3453 28768629 PMC5539518

[30] O'Callaghan G Fahy M Murphy P : Effectiveness of interventions to support the transition home after acute stroke: a protocol for a systematic review. 2021. 10.5281/zenodo.5145362 PMC8844528 35224442

