# Supplementary material for: Effectiveness of interventions to support the transition home after acute stroke: a protocol for a systematic review
Source: HRB Open Res. 2022 Mar 11;4:105. Originally published 2021 Sep 23. [Version 2] doi: 10.12688/hrbopenres.13364.2 (PMC8844528; doi:10.12688/hrbopenres.13364.2)
Supplement: Peer review history

# Peer review: Reviewer response for version 1

**DOI:** 10.21956/hrbopenres.14553.r31402

**Published:** 2022-02-14

**Authors:** Lena Von Koch, Sebastian Lindblom

**Affiliations:** 1 Department of Neurobiology, Care Sciences and Society, Division of Primary Health Care and Family Medicine, Karolinska Institutet, Stockholm, Sweden; 2 Department of Neurobiology, Care Sciences and Society, Karolinska Institutet, Stockholm, Sweden

**Stage 1: identifying the research question**

- Regarding “comparators, control”, the information of what “usual care” entails is often quite sparse. If available, we recommend the authors to include what “usual care” implied in the separate studies. This would strengthen the quality of the review and help readers interpret the findings.
- The primary outcome chosen is functional status, which is broad and unspecific. Could the authors please define and outline what will be included in functionals status?

Additional plausible secondary outcomes that would be of interest for outcomes of interventions to support transitions to home are patient satisfaction with care, specifically with the transition to home, measures on mood or wellbeing both on the part of the patient but also of caregivers, as well as their perceived care giver burden.

**Stage 3: Study selection**

- If available, it might be of interest to consider extracting the characteristics of professionals involved in the intervention. (Professions, competence, experience)
- The systematic review aims to explore the effectiveness of support interventions provided at transitions of care after stroke. However, the mechanism that drive such interventions successfully remains to a large extent unknown. Hence, if available, it would be of interest to extract suggested mechanisms and facilitators.

**Stage 5: Collating, summarising, and reporting the results**

- The inclusion criteria of different interventions are quite broad i.e., both different components and in different contexts, which will make the synthesising evidence challenging. How will this be handled in the analysis?

Is the study design appropriate for the research question?

Yes

Is the rationale for, and objectives of, the study clearly described?

Yes

Are sufficient details of the methods provided to allow replication by others?

Yes

Are the datasets clearly presented in a useable and accessible format?

Yes

Reviewer Expertise:

Sebastian Lindblom PhD: Neurorehabilitation, integrated care and care transitions

We confirm that we have read this submission and believe that we have an appropriate level of expertise to confirm that it is of an acceptable scientific standard.

# Response

**Published:** 2022-03-01

**Authors:** Geraldine O'Callaghan

**Affiliations:** Royal College of Surgeons in Ireland, Ireland

On behalf of all the protocol authors I would like to acknowledge the feedback that you have provided for our protocol – ‘Effectiveness of interventions to support the transition home after acute stroke; a protocol for a systematic review’.  We are extremely grateful to you for giving of your time and expertise, and hope the attached responses address your concerns and comments.

**1. Stage 1: identifying the research question.** **Regarding “comparators, control”, the information of what “usual care” entails is often quite sparse. If available, we recommend the authors to include what “usual care” implied in the separate studies. This would strengthen the quality of the review and help readers interpret the findings.**

 The authors agree that it is important to extract the available information on what denotes “usual care” in each study and we will do so in our systematic review.

**2. The primary outcome chosen is functional status, which is broad and unspecific. Could the authors please define and outline what will be included in functional status?**

 Functional status will be classified as per the activities component in the ICF Framework, i.e. the execution of a task or action by an individual, and the difficulties an individual may have in performing tasks of daily living (e.g. washing, dressing, walking around the house, preparing a meal).

**3. Additional plausible secondary outcomes that would be of interest for outcomes of interventions to support transitions to home are patient satisfaction with care, specifically with the transition to home, measures on mood or wellbeing both on the part of the patient but also of caregivers, as well as their perceived care giver burden.**

 All clinical outcomes including satisfaction with stroke care will be considered, as will caregiver outcomes of mood, quality of life, caregiver burden, and other outcomes included by the study researchers.   

**4. Stage 3: Study selection.** **If available, it might be of interest to consider extracting the characteristics of professionals involved in the intervention. (Professions, competence, experience).** **The systematic review aims to explore the effectiveness of support interventions provided at transitions of care after stroke. However, the mechanism that drive such interventions successfully remains to a large extent unknown. Hence, if available, it would be of interest to extract suggested mechanisms and facilitators.**

 The authors agree that increasing our knowledge in relation to the mechanisms that drive successful support interventions is important.  To this end, we will extract all available details in relation to the background and expertise of the facilitators in each study, and the methods used to deliver key strategies within each intervention.

**5. Stage 5: Collating, summarising, and reporting the results.** **The inclusion criteria of different interventions are quite broad i.e., both different components and in different contexts, which will make the synthesising evidence challenging. How will this be handled in the analysis?**

 Where possible, sub-group analysis according to patient (e.g. age, gender) or trial characteristics (e.g. recruitment setting, duration of intervention, length of follow-up, presence of caregiver) will be conducted.  This will address the breadth of the inclusion criteria and allow the authors to explore differences in response to an intervention.

# Peer review: Reviewer response for version 1

**DOI:** 10.21956/hrbopenres.14553.r30535

**Published:** 2021-10-19

**Authors:** Gert Kwakkel

**Affiliations:** 1 Department of Rehabilitation Medicine, Amsterdam Neuroscience and Amsterdam Movement Sciences, Amsterdam University Medical Centre, Amsterdam, The Netherlands

This is an important protocol for a systematic review of RCTs, cluster-RCTs and quasi randomized clinical trials investigating the effectiveness of types of supports provided at transitions of care. The Protocol is registered following PROSPERO and in line with statements of PRISMA.

 The systematic review is straightforward however I have some minor concerns.

1. Defining outcome functional status is broad and with that not clear. I suggest to further classify this following ICF in subdomains related with basic ADLs, extended ADLs, mobility.
2. Second outcomes: I suggest to include caregivers QoL including mood as well.
3. Stage 4 is clear. Indeed wise to use Rayyan software for search as well.
4. With respect to pooling of data. Please clarify the condition when pooling will be applied. How are the authors dealing with cluster randomized clinical trials in their pooling? When do the authors judge if the outcome (eg basic ADLs) can be pooled?

Is the study design appropriate for the research question?

Yes

Is the rationale for, and objectives of, the study clearly described?

Yes

Are sufficient details of the methods provided to allow replication by others?

Yes

Are the datasets clearly presented in a useable and accessible format?

Yes

Reviewer Expertise:

Stroke, behavioral Recovery, Neurorehabilitation, Systematic Reviews.

I confirm that I have read this submission and believe that I have an appropriate level of expertise to confirm that it is of an acceptable scientific standard.

# Response

**Published:** 2022-03-01

**Authors:** Geraldine O'Callaghan

**Affiliations:** Royal College of Surgeons in Ireland, Ireland

On behalf of all the protocol authors, I would like to acknowledge the feedback that you have provided for our protocol – ‘Effectiveness of interventions to support the transition home after acute stroke; a protocol for a systematic review’.  We are extremely grateful to you for giving of your time and expertise and hope the attached responses address your concerns and comments.

**1. Defining outcome functional status is broad and with that not clear. I suggest to further classify this following ICF in subdomains related with basic ADLs, extended ADLs, mobility.**

 Functional status will be classified as per the activities component in the ICF Framework, i.e. the execution of a task or action by an individual, and the difficulties an individual may have in performing tasks of daily living (e.g. washing, dressing, walking around the house, preparing a meal).

**2. Secondary outcomes: I suggest to include caregiver's QoL including mood as well.**

 The authors acknowledge that caring for stroke patients is challenging and may influence several subjective and objective aspects of the caregivers’ life.  In response to peer reviewer and patient and public involvement feedback, caregiver outcomes (e.g. QOL, mood, burden) will be included as measures of effectiveness of supports provided at the transition from hospital to home.

**3**. **Stage 4 is clear. Indeed wise to use Rayyan software for search as well.**

 Rayyan identify a number of shortfalls in its software capabilities i.e. RIS exports from a number of databases fail to import successfully into Rayyan, while duplicate detection, resolution, and reporting is still under development.  For this reason, the authors will export all search results into Endnote, de-duplicate in Endnote, and then export the remaining results to Rayyan for screening.

**4. With respect to pooling of data. Please clarify the condition when pooling will be applied. How are the authors dealing with cluster randomized clinical trials in their pooling? When do the authors judge if the outcome (e.g. basic ADLs) can be pooled.**

 Data will be pooled if the elements being pooled together are homogeneous, with respect to the parameters being estimated. Specifically, this means that, if there is similarity in terms of intervention variances then study data can be pooled i.e. if the interventions components and follow up time points are similar, and the same or comparable tool is used to measure the outcome (e.g. Barthel Index for ADLs), then it is fine to pool the data.

 The authors will identify any cluster-randomised trials in the review and explicitly state that they will conduct sensitivity analyses to investigate the robustness of meta-analysis findings based on their inclusion.
